# Effects of Transducer Placement on Load–Velocity Relationships in Smith Machine and Free Weight Squats in Trained Women

**DOI:** 10.3390/jfmk10020178

**Published:** 2025-05-15

**Authors:** Athanasios Tsoukos, Gregory C. Bogdanis

**Affiliations:** School of P.E. and Sport Science, National and Kapodistrian University of Athens, 17237 Athens, Greece; gbogdanis@phed.uoa.gr

**Keywords:** velocity-based training, biomechanics, strength assessment, countermovement jump, squat jump, drop jump

## Abstract

**Background**: We examined the effects of linear position transducer placement during Smith machine (SM) and free weight (FW) full squats on the mean velocity and the load–velocity relationship in trained women. In addition, we examined the relationship between the load–velocity characteristics and jump performance, to determine which testing approach is more appropriate for both the testing and transfer of training effects. **Methods**: Eleven trained women were assessed for 1-RM in FW and SM full back squats. Linear position transducers (LPTs) were attached to the barbell (BAR) and to the belt (BELT) during FW and SM full back squats. The mean velocity was measured across progressively increasing loads (30–100%). The load–velocity relationships were modeled using linear regression, and the velocity values, as well as the load–velocity parameters, were compared across all conditions (SM BAR, SM BELT, FW BAR, and FW BELT). Squat jump, countermovement jump, and drop jump performance were also assessed using an optical measurement system. **Results**: In SM compared to FW, 1-RM was higher (92.9 ± 16.2 kg vs. 85.1 ± 14.5 kg, *p* < 0.05, d = 0.53). A strong agreement was observed between the FW BAR and FW BELT (Lin’s concordance correlation coefficient CCC = 0.96–0.99), as well as between the SM BAR and FW BAR (CCC = 0.95–0.97) at low-to-moderate intensities (30–70% 1-RM), suggesting that these conditions can be used interchangeably. However, the SM BELT systematically showed lower mean velocity values at 30–80% 1-RM and exhibited low agreement across all other conditions. In contrast, the FW BELT mean velocity was lower than that of the FW BAR and SM BAR only at higher intensities (>80% 1-RM). V0 and mean velocities at low-to-moderate loads (30–70% 1-RM) showed strong correlations with all jump types, with relationships gradually weakening as the load increased (r = 0.63–0.93, *p* < 0.05). The highest correlations were observed in the SM BAR and FW BELT conditions. Lastly, the relative strength demonstrated a consistent relationship with squat jump and drop jump performance exclusively in the FW condition (r = 0.71 and 0.72, *p* < 0.05). **Conclusions**: The FW BAR and FW BELT showed strong agreement at submaximal loads and may be used interchangeably, while the SM BELT showed a lower mean velocity and low agreement with other conditions. The load–velocity relationship parameters and mean velocity at low-to-moderate loads correlated strongly with the jump performance. Coaches and practitioners can use bar-mounted and belt-mounted LPTs interchangeably during FW squats for velocity-based training at submaximal intensities when working with trained women. Additionally, tracking the mean velocity at low-to-moderate loads provides valuable insights into lower-body explosive performance, supporting more precise and individualized training prescriptions and performance monitoring.

## 1. Introduction

During resistance training, strength and conditioning coaches and athletes utilize either free weights (FW) or a Smith machine (SM) to improve muscular strength [1]. In FW exercises, the barbell is allowed to move freely, demanding greater stabilization from the lifter [2], whereas SM provides a guided bar path, enhancing balance and potentially altering neuromuscular activation patterns and force production. Research indicates that in women, maximum strength, measured as the one-repetition maximum (1-RM), is 7.7% greater in the SM squat compared to the FW squat [3], and nearly 7% greater during the Bulgarian split squat exercise [4]. In exercises requiring less stabilization, such as the bench press exercise, the results are inconsistent, with studies reporting either higher or lower 1-RM strength in FW compared to SM [3,5]. Similarly, when examining movement velocity, some studies report higher mean and peak velocities when machine-based versions of the power clean are used, compared to FW [6], whereas other studies report higher velocities in FW compared to SM in exercises such as bench press, full squat, shoulder press, and prone bench pull [7]. These differences in force production and movement velocity may be attributed to differences in the barbell’s trajectory [6] or variations in the activation of the muscles involved. FW exercises typically elicit greater electromyographic activity (EMG) in stabilizing muscles during the bench press and in prime movers during the squat [2,8]. Despite these differences in force production and movement velocity, recent research showed that both methods induced similar muscle architectural adaptations and comparable load–velocity-profile characteristics [1].

The load–velocity relationship (L-V relationship) is an inverse linear association between load and movement velocity. It is commonly used for one-repetition maximum (1-RM) prediction, monitoring relative intensity, identifying deficiencies in the load–velocity profile, and prescribing individualized training programs based on velocity-based training principles [9,10,11,12]. The barbell velocity is typically measured using sensors such as linear position transducers, which are typically attached to the barbell [13,14,15]. However, most of these sensors measure movement velocity with high accuracy on a SM because the barbell follows a fixed vertical path, limiting the movement to a single dimension [16,17,18]. In contrast, during FW training, the barbell moves in both vertical and horizontal planes, and if a linear position transducer is used, it may introduce measurement errors when recording the movement velocity [13]. In the squat exercise, these errors can be controlled, and accuracy can be improved through the consistent placement of the device relative to the position of the participant, as well as by establishing proper exercise form and technique [13].

Another solution to limit horizontal displacement and increase the accuracy of measurement is to attach the sensor to the body (e.g., a belt) during the squat. Research has shown that the barbell exhibits greater movement than the center of mass, and as such, velocity measurements obtained from the barbell may be overestimated when compared to those measured from the center of mass [19,20,21]. These discrepancies can be reduced by using sensors attached to the body or belt, providing more accurate velocity data [22]. Therefore, it would be valuable to examine the L-V relationship in the commonly used squat exercise on the SM and using FW, by attaching a linear position transducer either to the barbell or to a belt worn by the participant.

The load or force–velocity profile characteristics have been previously correlated with jump performance [23,24]. For instance, Baena-Raya et al. [23] found that L-V relationship variables were significantly associated with block and spike jump height, as well as jumping spike speed in elite male volleyball players. Similarly, Yamauchi et al. [24] reported that maximum force and power were positively correlated with jump performance. Given this background, it would be valuable to investigate whether the relationships between the load–velocity characteristics and jump performance are stronger when using load–velocity data from the belt or barbell under SM and FW conditions.

Considering the above and the lack of research comparing the L-V relationships between SM and FW in women using sensors attached to both the belt and the barbell, the purpose of the present study was to examine the movement velocity differences between these modalities using two linear position transducers (barbell- and belt-mounted sensors), and their effect on the L-V relationship. Additionally, we examined the relationship between the load–velocity characteristics and countermovement jump, squat jump, and drop jump performance, and assessed their agreement, interchangeability, and concurrent validity. Recent studies focusing on velocity-based training in female populations have provided valuable insights into sex-specific adaptations and training responses, highlighting the importance of further exploring these adaptations to optimize performance outcomes in women [25,26,27,28,29,30,31]. This study aims to address the research gap by providing insights into the practicality and effectiveness of using barbell- and belt-mounted sensors for velocity-based training in trained women, ultimately contributing to more efficient training strategies for female athletes.

## 2. Materials and Methods

### 2.1. Participants

An a priori sample size calculation using G*Power (v. 3.1.9.7) was performed with a medium effect size (Cohen’s d = 0.50), an alpha level of 0.05, a statistical power of 0.80, and a correlation among repeated measurements of 0.7 [3]. This calculation suggested that a sample size of 10 participants would be sufficient to detect significant effects. The actual statistical analysis revealed a η^2^p of 0.75 (see Section 3.3), indicating a large effect. This suggests that the effects observed were stronger than anticipated based on the prior power analysis, and that the sample size in the present study was adequate to detect these larger than expected effects.

Eleven female track and field athletes participated in the study (age: 22.6 ± 1.5 years, height: 1.66 ± 0.07 m, body mass: 61.9 ± 4.8 kg). All participants had a minimum of three years of experience in strength and power training and specialized in sprinting (100–400 m), jumping (long jump and triple jump), and throwing events (javelin throwing) at national and international levels. The inclusion criteria for participation were as follows: (a) a minimum of three years of consistent resistance training (including squats performed at least two times per week with FW, although experience with the SM was comparatively lower), (b) no musculoskeletal injuries in the past year, and (c) no use of nutritional supplements or performance-enhancing drugs. Before participation, all athletes received a detailed explanation of the testing protocol, possible risks, and their right to withdraw at any time without providing an explanation. Written informed consent was obtained from each participant. The study was approved by the local Institutional Review Board (Approval no. 1699/18-11-2024) and was conducted in accordance with the ethical principles of the World Medical Association (Helsinki declaration of 1964, revised in 2013).

### 2.2. Research Design

A repeated-measures design was employed to investigate differences in the mean velocity between SM and FW using a sensor attached either to the barbell or the belt. The secondary purpose was to examine the relationship between the load–velocity characteristics and countermovement jump (CMJ), squat jump (SJ), and drop jump (DJ), while also assessing their agreement and interchangeability. The study included two experimental conditions (FW and SM), arranged in a randomized and counterbalanced order, with one-week intervals between sessions. In both conditions, participants first completed a general warm-up, followed by dynamic stretches and two specific warm-up sets of six full squat repetitions performed at submaximal movement velocity with loads of 20 kg and 30 kg. Afterwards, they undertook a one-repetition maximum (1-RM) test as previously described [32,33]. The mean velocity was recorded for each repetition during the 1-RM test. The within-session reliability results are presented in Table 1. Within-session reliability was assessed by analyzing the repeated measurements obtained from at least two separate repetitions performed at each load, as participants completed two sets of 1–2 repetitions per load (except the 1-RM load). In the FW condition, the mean velocity was assessed for both the barbell (FW-BAR) and the belt (FW-BELT). Similarly, in the SM condition, the mean velocity was recorded for the barbell (SM-BAR) and the belt (SM-BELT).

### 2.3. Maximum Dynamic Strength (1-RM) in the Full Squat Exercise

Maximum dynamic strength (one-repetition maximum: 1-RM) was assessed using both the Smith machine (SM) and a squat rack in the FW condition with an Olympic barbell (20 kg) and weight plates, following the procedures outlined by the National Strength and Conditioning Association (NSCA) [34]. Participants regularly performed full (deep) squats as part of their training routine and were highly experienced with the exercise. Every repetition was executed as fast as possible during the upward phase (concentric), while during the downward (eccentric) phase, participants were instructed to control the movement. In the FW condition, the participants adopted a shoulder-width foot stance, maintained a slightly arched back, and kept their feet flat on the floor [35,36]. In the SM condition, participants followed the same guidelines as previously outlined [37].

A repetition was considered successful when the thigh dropped below the parallel position [35,36,38]. The knee angle at the lowest point of the eccentric phase was measured using an iPhone (Apple Inc., Cupertino, CA, USA) at 60 fps, with the Kinovea Video Analysis Software (v. 0.8.15). Assessment of the knee angle was conducted to verify that participants performed the squats within the appropriate and standardized range of motion [38]. The validity and reliability of this procedure has been shown elsewhere [39,40]. To ensure safety and provide motivation, two experienced spotters, both strength and conditioning coaches, were present to offer verbal encouragement throughout the assessment. The initial load was set at 30% of the predicted 1-RM based on the previous 1-RM tests that the participants had performed during training. The participants then performed five to eight incremental loads until reaching their 1-RM. Rest intervals between sets were 3–5 min to ensure adequate recovery and minimize fatigue between attempts.

### 2.4. General and Specific Warm-Up

Participants underwent a standardized warm-up protocol including five minutes of light cycling (at a resistance of 50–60 W) followed by five minutes of dynamic stretches targeting the muscles of the legs and the lower back [33,40]. After the general warm-up, the participants performed a specific warm-up, which consisted of two sets of six repetitions full squats at 20 kg and 30 kg with controlled movement velocity. The recovery time between these sets was 3 min.

### 2.5. Sensor Placement

Two linear position transducers (Tendo Power Analyzer System v. 314, TENDO Sports Machines, Trencin, Slovak Republic) were placed on the floor to measure the mean velocity during the full squat. One transducer tracked the barbell and the other the athlete’s waist via a belt [41]. The linear position transducer measuring the barbell movement had its string attached to the right side of the shaft, two (2) cm from the bearings/brushings, ensuring a stable attachment point while minimizing potential interference from the athlete’s arm. The second linear position transducer, tracking the athlete’s body movement, had its string secured to a belt at the athlete’s waist, aligned with the sagittal plane. The transducers were placed so that the strings were vertical when the participant was standing upright before starting the movement.

### 2.6. Jump Performance

The SJ, CMJ, and DJ performance were assessed using an optical measurement system (Optojump Next; Microgate, Bolzano, Italy), which recorded the flight and contact times [42,43]. For the SJ, participants stood with their feet shoulder-width apart and hands on the hips. They lowered into a squat until reaching a 90 degree knee angle, held the position for 3 s, and then jumped as high as possible [44]. During the CMJ, athletes performed maximal vertical jumps while keeping their hands on their hips, ensuring a consistent body position during take-off and landing [45,46,47,48]. For the DJ, participants stood on a 15 cm box with their feet hip-width apart at the edge [49]. They stepped off passively, allowing gravity to dictate the descent without any active propulsion. Upon landing on the balls of their feet, they immediately executed a maximal vertical jump, aiming to minimize ground contact time. Throughout the movement, their hands remained on their hips to standardize arm involvement [47]. Each participant completed two attempts, with 30 s of rest between trials, and the best value was kept for statistical analysis. The jump performance was evaluated after the general warm-up of the first main trial.

### 2.7. Statistical Analysis

Statistical analyses were conducted using the STATISTICA v.12.0 software (StatSoft, Inc., Tulsa, OK, USA). The data are presented as means ± standard deviations (SDs). Dependent *t*-tests were performed to assess differences in the maximum and relative strength, as well as the knee angle, between the SM and FW. The L-V relationships were modeled using linear regression. Based on this, we calculated the V0 (maximum theoretical velocity), L0 (maximum theoretical load), slope, standard error of estimate (SEE), and Pearson’s correlation. A three-way repeated-measures ANOVA (2 methods [SM and FW] × 2 points of attachment [bar and belt] × 8 loads) was conducted to examine differences in the mean velocity across conditions and loads (Conditions: FW and SM x Points of attachment: BELT and BAR x Loads: 30%, 40%, 50%, 60%, 70%, 80%, 90%, and 100%). Additionally, a two-way repeated-measures ANOVA was performed to assess differences in the V0, L0, and slope. When significant main effects or interactions were detected, post hoc pairwise comparisons were conducted using Tukey’s HSD. The effect sizes for main effects and interactions were evaluated using partial eta squared (η^2^p), classified as small (0.01 to 0.059), moderate (0.06 to 0.137), and large (>0.137). For pairwise comparisons, Cohen’s d was used, classified as small (0.2), medium (0.5), and large (0.8). Agreement and validity were assessed using Pearsons’s correlations, typical error (TE), and Bland–Altmann plots. Lin’s concordance correlation coefficient (CCC) was also used to evaluate the concurrent validity between the sensor attachment methods and between the SM and FW. The CCC strength was interpreted as almost perfect (>0.99), substantial (0.95–0.99), moderate (0.9–0.95), and poor (<0.90). To compare correlation coefficients across conditions, Pearson’s r values were transformed into Fisher’s Z scores. A one-way repeated-measures ANOVA was performed on the Fisher’s Z-transformed values to assess differences in the correlation strength across conditions. Also, Pearson’s correlation coefficients were calculated to examine the relationships between the jump height in CMJ and SJ, as well as RSI (during DJ), and the mean velocities recorded across all loads and all conditions. Statistical significance was set at *p* < 0.05.

## 3. Results

### 3.1. Maximum and Relative Strength

Dependent *t*-tests revealed significantly higher maximum and relative strength in the SM condition compared to using FWs (Table 2). However, no significant difference was observed in knee angle between the two conditions (Table 2).

### 3.2. Load–Velocity Parameters and Pearson’s Correlations Across Conditions

A two-way ANOVA revealed a significant effect for V0 (*p* < 0.001, η^2^p = 0.68). Tukey’s post hoc tests showed that V0 on the Smith machine with the sensor on the belt (SM BELT: 1.53 ± 0.19 m∙s^−1^) was significantly lower (*p* < 0.01) compared to all other conditions (SM BAR: 1.62 ± 0.19 m∙s^−1^, d = 0.51; FW BAR: 1.64 ± 0.20 m∙s^−1^, d = 0.62; FW BELT: 1.65 ± 0.21 m∙s^−1^, d = 0.64) (Table 3). No significant differences were observed between the SM BAR, FW BAR, and FW BELT (*p* > 0.05).

For L0, two-way ANOVA was also significant (*p* = 0.004, η^2^p = 0.58). Tukey’s post hoc tests indicated that L0 on the SM (SM BAR: 118.3 ± 20.3 kg and SM BELT 119.8 ± 21.1 kg) was significantly higher (*p* < 0.01) than both FW points of the sensor attachment (FW BAR: 109.6 ± 16.7 kg, d = 0.49 and 0.56 from the SM BAR and SM BELT; FW BELT: 107.9 ± 16.8 kg, d = 0.59 and 0.65 from the SM BAR and SM BELT) (Table 3). No significant differences were found between attachment points within the Smith machine (SM BAR and SM BELT; *p* > 0.05) or within the free weight attachment points (FW BAR and FW BELT; *p* > 0.05).

A significant interaction effect was observed for the slope (*p* < 0.001, η^2^p = 0.73). Tukey’s post hoc tests showed that the slope on the SM (SM_BAR: −0.014 ± 0.003 m∙s^−1^∙kg^−1^ and SM BELT: −0.013 ± 0.003 m∙s^−1^∙kg^−1^) differed significantly (*p* < 0.01) from both FW conditions (FW BAR: −0.015 ± 0.003 m∙s^−1^∙kg^−1^ d = 0.44 and 0.81 from the SM BAR and SM BELT; FW BELT: −0.015 ± 0.003 m∙s^−1^∙kg^−1^ d = 0.54 and 0.92 from the SM BAR and SM BELT). No significant differences were found between the FW BAR and FW BELT (*p* > 0.05). However, a small but significant difference was observed between the SM BAR and SM BELT (*p* = 0.001, d = 0.34).

For SEE, two-way ANOVA interaction was not significant (*p* = 0.95, η^2^p = 0.0004), suggesting that SEE was consistent across conditions (SM and FW and BAR and BELT), with no differences in the prediction error based on the attachment point or type of squat used. Additionally, no main effects were observed either (*p* > 0.63). Similarly, two-way ANOVA interaction was not significant for Pearson’s r (*p* = 0.32, η^2^p = 0.10), indicating that Pearson’s r was consistent across the conditions (SM and FW and BAR and BELT), and that the strength of the linear relationship remained unchanged regardless of the type of squat or attachment point. Likewise, the main effects were not significant (*p* > 0.32).

### 3.3. Comparison of Mean Velocity Across Conditions and Sensor Attachment Points

Three-way ANOVA revealed a significant interaction between the conditions and loads for the mean velocity (*p* < 0.0001, η^2^p = 0.75). Tukey’s post hoc tests showed that the mean velocity was significantly lower as the load increased in all conditions and sensor attachment points (*p* < 0.001). Tukey’s post hoc comparisons between the type of squat and sensor attachment points showed that the mean velocity in the SM BELT was significantly different from that in the SM BAR at 30% to 90% loads (*p* < 0.05, d = 0.36–0.49), from that in the FW BAR at 30% to 80% loads (*p* < 0.05, d = 0.46–0.66), and from that in the FW BELT at 30% to 70% and 100% loads (*p* < 0.05, d = 0.32–0.52) (Table 3). The mean velocity in the SM BAR was significantly different from that of the FW BAR at 30% and 40% loads, but with a very small effect size (*p* < 0.05, d = 0.12 and 0.12), and at 30%, 90%, and 100% compared to the FW BELT (*p* < 0.05, d = 0.10, 0.40, and 0.52). Lastly, the mean velocity in the FW BAR differed significantly compared to the FW BELT from 80% to 100% loads (*p* < 0.05, d= 0.25, 0.33, and 0.35).

### 3.4. Agreement and Interchangeability Analysis Between SM BAR and FW BAR

As mentioned above, the three-way ANOVA did not show a significant difference in the mean velocity between the SM BAR and FW BAR, except for the loads 30% and 40% (Table 4); however, the effect was very small (d = 0.12 for both loads). Lin’s CCC indicated very strong agreement between FW and SM from 30% to 70% of 1-RM (r_c_ = 0.95–0.97). However, agreement weakened at higher loads, with moderate-to-poor correlations at 80% (r_c_ = 0.88), 90% (r_c_ = 0.71), and 100% (r_c_ = 0.57), suggesting increased variability at maximal intensities (Table 5). The typical error for mean velocity differences ranged from 0.01 to 0.03 m∙s^−1^ (1.6–8.7%). Finally, the Bland–Altman plots confirmed that all differences in the mean velocity remained within the 95% limits of agreement, supporting excellent consistency in the absolute measurements across all loads (Appendix A.1).

### 3.5. Agreement and Interchangeability Analysis Between SM BELT and FW BELT

The three-way ANOVA revealed significant differences in the mean velocity between the SM BELT and FW BELT across loads from 30% to 70%, with the SM BELT consistently showing lower values (Table 4). Lin’s CCC indicated moderate-to-poor correlations (Table 5), suggesting that the agreement between the two devices was limited in terms of their relative measurements, with greater variability observed at maximal intensities. The typical error (TE) for the mean velocity differences ranged from 0.02 to 0.04 m∙s^−1^ (2.6–7.8%). The Bland–Altman plots further supported these findings, confirming that the differences in the mean velocity remained within the 95% limits of agreement, indicating good consistency in the absolute measurements across all loads. However, for loads between 30 and 70%, the mean velocity was lower in the SM BELT and showed systematically lower values, suggesting a potential systematic bias in the mean velocity measurements compared to the FW BELT (Appendix A.2).

### 3.6. Agreement and Interchangeability Analysis Between SM BAR and SM BELT

The three-way ANOVA revealed significant differences in the mean velocity between the SM BAR and SM BELT across loads from 30% to 90%, with the SM BELT consistently showing lower values (Table 4). Lin’s CCC indicated moderate-to-poor correlations (Table 5), suggesting that the agreement between the two devices was limited in terms of their relative measurements. The typical error (TE) for the mean velocity differences ranged from 0.01 to 0.03 m∙s^−1^ (2.8–3.1%). The Bland–Altman plots showed that the differences in the mean velocity remained within the 95% limits of agreement, indicating good consistency in the absolute measurements across all loads. However, for loads between 30 and 90%, the mean velocity in the SM BELT was consistently lower compared to in the SM BAR (Appendix A.3.).

### 3.7. Agreement and Interchangeability Analysis Between FW BAR and FW BELT

The three-way ANOVA revealed no significant differences in the mean velocity between the FW BAR and FW BELT across loads, except the higher intensities from 80% to 100%, where the FW BELT consistently showed lower values (Table 4). Lin’s CCC indicated a very strong agreement for relative measurements between the FW BAR and FW BELT from 30% to 70% of 1-RM (r_c_ = 0.96–0.99). However, the agreement weakened at higher loads, with moderate correlations at 80% (r_c_ = 0.93), 90% (r_c_ = 0.90), and 100% (r_c_ = 0.89), suggesting a slight increase in variability at maximal intensities (Table 5). The typical error (TE) for the mean velocity differences ranged from 0.011 to 0.013 m∙s^−1^ (1.0–3.7%). The Bland–Altman plots further supported these findings, confirming that the differences in the mean velocity remained within the 95% limits of agreement, indicating good consistency in the absolute measurements across all loads. However, for loads between 80 and 100%, the mean velocity was consistently lower in the FW BELT compared to the FW BAR (Appendix A.4.).

### 3.8. Relationships of Jump Performance with Load–Velocity Parameters and Strength Measures in SM and FW

Table 6 presents the relationships between the jump performance and load–velocity parameters, as well as strength measures. Among all conditions, only V0 exhibited a strong and significant correlation with the jump performance (r = 0.74–0.93; *p* < 0.05). Regarding strength measures, only relative strength in free weights showed a strong association with the SJ and DJ (r = 0.70 and 0.71; *p* < 0.05).

### 3.9. Relationships of Jump Performance with SM BAR Velocities Across All Loads

The jump performance showed strong relationships between all type of jumps and bar velocities from low-to-moderate loads, with correlations gradually decreasing as the load increased (Table 7). Notably, at a 100% load, the correlations were not statistically significant.

### 3.10. Relationships of Jump Performance with SM BELT Velocities Across All Loads

The jump performance demonstrated strong correlations with belt velocities across all jump types at low-to-moderate loads, with these relationships gradually weakening as the load increased (Table 7). Notably, at 90% and 100% loads, the correlations were not statistically significant, except for the DJ. Additionally, the correlations observed in the SM BELT condition between the belt velocities and jump performance were generally lower compared to the corresponding relationships with bar velocities.

### 3.11. Relationships of Jump Performance with FW BAR Velocities Across All Loads

The jump performance showed strong relationships between all types of jumps and bar velocities from low-to-moderate loads, with correlations gradually decreasing as the load increased (Table 7). Notably, at 90% and 100% loads, the correlations were not statistically significant. Additionally, the correlations observed in the FW BAR condition between the bar velocities and jump performance were generally higher compared to in the SM BELT condition.

### 3.12. Relationships of Jump Performance with FW BELT Velocities Across All Loads

The jump performance showed strong relationships between all types of jumps and belt velocities from low-to-moderate loads, with correlations gradually decreasing as the load increased (Table 7). Notably, at 90% and 100% loads, the correlations were not statistically significant. Additionally, the correlations observed in the FW BAR condition between the bar velocities and jump performance were generally higher compared to in the SM BELT condition and comparable to the SM BAR condition.

## 4. Discussion

The main finding of the present study was that there was a strong agreement between the FW BAR and FW BELT, as well as between the SM BAR and FW BAR at low intensities (30–70% 1-RM), suggesting that these testing setups may be used interchangeably in women. In contrast, the mean velocity was lower in the SM BELT, and there was also lower agreement with the other conditions. The load–velocity relationship parameters, such as V0, as well as the mean velocity at low-to-moderate loads, correlated strongly with the jump performance, with the highest correlations observed in the SM BAR and FW BELT conditions. These findings indicate the strong concurrent validity of the L-V relationship in assessing explosive performance, particularly at submaximal loads. Lastly, the relative strength demonstrated a consistent relationship with the SJ and DJ performance exclusively in the FW condition.

In the present study, the mean velocity was lower when the sensor was attached to the BELT compared to the FW, particularly in the SM BELT at low and moderate loads and the FW BELT at heavier loads (>80% 1-RM). Previous research has shown that the barbell is displaced 13.4% more than the body’s center of mass, and its velocity is approximately 16.1% greater [19]. Similarly, McBride et al. [20] reported that the barbell travels a greater distance than the body, resulting in a higher peak velocity for the bar compared to the body’s center of mass. However, in the present study, the differences in the mean velocity were not as pronounced or consistent across all loads as those reported in previous studies. A possible explanation for this discrepancy is the difference in measurement techniques. The aforementioned studies utilized sophisticated 3D kinetic and kinematic systems, which are considered more accurate but also significantly more expensive. In contrast, our study employed a low-cost yet reliable linear position transducer [13,41,50,51,52], which, despite its limitations, provides a practical alternative for velocity-based training assessments.

A very strong linear association was found between the lifted load and the mean velocity across all conditions (r > −0.983), with no significant differences between conditions (*p* > 0.05). This indicates that the L-V relationship is highly consistent and provides strong predictability across all conditions. These results align with findings from other studies [10,53,54,55,56]. The standard error of estimate (SEE) values in the present study ranged from 0.049 to 0.055 m∙s^−1^, which is relatively low compared to other studies [54,57]. This low SEE may be attributed to the fact that the female participants in our study were trained athletes, possessing high proficiency in the squat technique and performing the movement with good stability. Additionally, the slope of the L-V relationship was steeper in the FW conditions compared to SM conditions. Specifically, the slopes in the FW conditions were −0.015 m∙s^−1^∙kg^−1^, while the slopes using the SM were slightly shallower, ranging from −0.013 to −0.014 m∙s^−1^∙kg^−1^. This suggests that the mean velocity decreased more rapidly as the load increased in FW. The steeper slopes in the FW conditions may be due to the greater neuromuscular demands and less stability inherent in FW exercises, which could result in more force production variability across the L-V relationship [2,8]. In contrast, the fixed bar path in the SM provides more stability, which likely leads to more consistent force production and a less pronounced velocity decrease, resulting in a shallower slope [3,4].

In the present study, we evaluated the agreement and interchangeability between sensor placements and exercises using three-way ANOVA, Lin’s Concordance Correlation Coefficient (CCC) [58], and Bland–Altman plots. The comparison between the SM BAR and FW BAR showed very strong agreements across a wide range of loads (30–70% of 1-RM). These results were mirrored in the comparison between the FW BAR and FW BELT, indicating that both sensor placements produced similar mean velocity outcomes in the low-to-moderate intensity ranges. However, at higher loads (80–100% of 1-RM), the agreements between both the SM BAR vs. FW BAR and FW BAR vs. FW BELT weakened, suggesting greater variability at near-maximal intensities. This decrease in agreement at higher loads likely reflects the inherent challenges in measuring the velocity at maximal intensities, where small fluctuations in movement or sensor placement could introduce discrepancies. Despite these slight variations, both comparisons demonstrated excellent consistency in the absolute velocity measurements, with all sensors producing very similar results across most loads. Interestingly, the mean velocity was lower in the FW BELT compared to the FW BAR. In contrast, the SM BELT showed significant differences in the mean velocity when compared to both the SM BAR and FW BELT at a wide range of loads (30–70% of 1-RM). The SM BELT consistently recorded lower mean velocity values. This systematically lower mean velocity in the SM BELT could be attributed to the different movement of the hips when the bar follows a fixed path, leading to differences in the sensor’s recordings. The poorer-to-moderate agreement, as indicated by Lins’s CCC, further supports that the SM BELT’s sensor placement is not interchangeable with the other sensor attachments showing lower mean velocity values, particularly between 30 and 70% of 1-RM. Thus, while the SM BAR and FW BAR, as well as the FW BAR and FW BELT, demonstrated strong agreement and interchangeability across various loads, the SM BELT displayed unique characteristics that made it unsuitable for interchangeability with the other sensor placements. This finding underscores the importance of the sensor placement and exercise modality when interpreting velocity measurements in strength training settings.

In the present study, the SM BELT showed lower mean velocity values compared to the other transducer placements. This result may be attributed to the altered hip biomechanics by the SM during squats. The fixed vertical bar path of the SM constrains natural forward–backward movement [59], encouraging a more upright trunk position and reducing the involvement of the posterior chain. This leads to decreased hip flexion and anterior–posterior hip displacement. As a result, the distribution of joint torques—especially at the hip and lumbosacral spine—is modified [59], which may explain the lower velocity values recorded by the belt-mounted transducer in this condition.

One important finding of the present study was that the jump performance exhibited strong correlations with the V0 and mean velocities at low-to-moderate loads, with these relationships gradually weakening as the load increased. The strongest correlations were observed in the SM BAR and FW BELT conditions, suggesting that the L-V relationship is a valid method for assessing explosive performance, particularly at submaximal loads. These findings align with previous research demonstrating moderate to strong relationships between the load–velocity or force–velocity parameters and power or jump performance [23,24,60,61,62]. This result reinforces the concept of velocity specificity in trained women, highlighting the critical role of V0 in vertical jump performance [63]. The stronger correlations at lower loads suggest that submaximal velocities may be more reflective of the load–velocity profile in performance assessment.

The finding that the maximum strength, relative strength, and maximum theoretical load (L0) were significantly higher using the SM compared to FW aligns with previous research indicating that the fixed bar path of the SM reduces stabilization demands, allowing lifters to focus more on force production [3,4,5]. Cotterman et al. [3] compared muscle force production using the SM and FW for bench press and squat exercises, reporting that the squat 1-RM was higher in the SM condition. When sex was considered in the analysis, women’s 1-RM was 7.7% higher using the SM than FW (SM: 86.6 ± 13.8 kg vs. FW: 80.4 ± 17.2 kg, *p* < 0.01), a finding comparable to our study, where the percentage difference was 9.2% (SM: 92.9 ± 16.2 kg vs. FW: 85.1 ± 14.5 kg, *p* < 0.01). These results suggest that if the primary goal of resistance training is to maximize load, the SM may be the preferred choice. However, research has shown greater activation of stabilizing muscles during FW exercises compared to the SM [2,8]. Therefore, the most effective training approach may be integrating both methods, using the SM to target maximum loading and FW to enhance stabilization and motor control.

The L0 and absolute strength did not correlate with the jump performance in the present study. This finding aligns with previous research indicating that the maximum isometric force, as L0 represents, does not strongly correlate with the jump height [64]. Similarly, a study on NCAA Division I-AA male football and track and field athletes has shown that the absolute maximum strength is not significantly related to the jump performance [65]. In contrast, multi-joint dynamic strength tests, such as 1-RM squat and 1-RM power clean, when expressed relative to body mass, are most closely associated with CMJ performance [65]. These findings are consistent with the results of the present study, which revealed significant relationships only with the relative strength, not with absolute strength, in trained women. This may be partly explained by a ceiling effect in our sample, as participants had likely already developed high levels of absolute strength, limiting further gains in jump performance through strength alone. In such situations, variables like relative strength, muscle power output, and rate of force development may play a more critical role in enhancing explosive performance [65].

This study has some limitations. First, the use of a linear position transducer, while reliable and cost-effective, may not capture kinematic data with the same accuracy as high-resolution motion analysis systems. Additionally, while the study included trained female athletes, the findings may not be directly generalizable to male athletes or untrained individuals, as neuromuscular demands and movement mechanics differ. Finally, the study primarily focused on the L-V relationship and its association with jump performance, but other variables, such as the rate of force development or muscle activation patterns, were not assessed and could provide further insights into training adaptations. Moreover, although the sample size of eleven participants may be considered relatively small for ensuring the robustness and generalizability of the results, both the a priori power analysis and the observed power of the ANOVA support the drawing of valid conclusions.

## 5. Conclusions

The FW BAR and FW BELT showed strong agreement at submaximal loads and may be used interchangeably, while the SM BELT showed lower mean velocity and low agreement with other conditions. The L-V relationship parameters and mean velocity at low-to-moderate loads correlated strongly with the jump performance and may be used by coaches for testing and training purposes. This range is particularly relevant for improving jump performance, as our findings suggest a strong relationship between the load–velocity characteristics and explosive lower-body actions. The relative strength demonstrated a consistent relationship with the SJ and DJ performance exclusively in the FW condition, suggesting that free weight training may have a greater transfer effect on explosive lower-body performance compared to the SM. When implementing velocity-based training, coaches should be aware of the effects of the type of squat (SM or FW) and linear position transducer placement on the load–velocity profiles.

## Figures and Tables

**Table 1:** Within-session reliability metrics for mean velocity measurements across different conditions. FW-BAR: mean velocity of the barbell in the FW condition; FW-BELT: mean velocity of the belt in the FW condition; SM-BAR: mean velocity of the barbell in the SM condition; and SM-BELT: mean velocity of the belt in the SM condition.

	FW-BAR	FW-BELT	SM-BAR	SM-BELT
ICC	0.987	0.986	0.993	0.992
LOWER	0.970	0.969	0.984	0.969
UPPER	0.994	0.993	0.997	0.993
SEM (m∙s^−1^)	0.022	0.024	0.013	0.014
% SEM	2.5%	2.7%	1.5%	1.7%

**Table 2:** Maximum and relative strength of the participants and knee angle differences between the free weight and Smith machine conditions. Values are presented as mean ± SD.

Variable	Free Weights	Smith Machine	Cohen’s D
Maximum Strength (kg)	85.1 ± 14.5	92.9 ± 16.2 *	0.53
Relative Strength (kg∙kg^−1^)	1.38 ± 0.23	1.50 ± 0.24 *	0.55
Knee Angle (°)	46.0 ± 11.5	45.1 ± 13.2	0.08

*: *p* < 0.01 from the free weights condition.

**Table 3:** Summary of load–velocity parameters for each condition and sensor attachment point. Pearson’s correlation coefficient r with Fisher’s Z-transformed values (mean ± SD).

Condition	V0 (m∙s^−1^)	L0 (kg)	SLOPE (m∙s^−1^∙kg^−1^)	SEE (m∙s^−1^)	Pearson’s r (Fisher’s Z-Transformed)
SM BAR	1.62 ± 0.19 *	118.3 ± 20.3 #	−0.014 ± 0.003 *#	0.050 ± 0.025	−0.988 (−2.8 ± 0.8)
SM BELT	1.53 ± 0.19	119.8 ± 21.1 #	−0.013 ± 0.003 #	0.049 ± 0.021	−0.986 (−2.6 ± 0.5)
FW BAR	1.64 ± 0.20 *	109.6 ± 16.7	−0.015 ± 0.003	0.055 ± 0.020	−0.983 (−2.5 ± 0.4)
FW BELT	1.65 ± 0.21 *	107.9 ± 16.8	−0.015 ± 0.003	0.054 ± 0.019	−0.985 (−2.5 ± 0.3)

*: *p* < 0.01 from SM BELT; #: *p* < 0.01 from FW BAR and FW BELT.

**Table 4:** Mean velocity across types of squat and sensor attachment points. Values are mean ± SD.

	Conditions			
Loads	SM BAR	SM BELT	FW BAR	FW BELT
30%	1.24 ± 0.13 *	1.18 ± 0.13	1.25 ± 0.14 *†	1.25 ± 0.15 *†
40%	1.11 ± 0.11 *	1.06 ± 0.11	1.12 ± 0.12 *†	1.12 ± 0.13 *
50%	0.98 ± 0.09 *	0.94 ± 0.10	0.99 ± 0.10 *	0.99 ± 0.11 *
60%	0.86 ± 0.08 *	0.82 ± 0.08	0.87 ± 0.08 *	0.86 ± 0.09 *
70%	0.73 ± 0.06 *	0.70 ± 0.06	0.74 ± 0.07 *	0.72 ± 0.07 *
80%	0.61 ± 0.05 *	0.58 ± 0.05	0.61 ± 0.05 *	0.59 ± 0.06 ‡
90%	0.48 ± 0.04 *	0.47 ± 0.04	0.48 ± 0.05	0.46 ± 0.05 †‡
100%	0.35 ± 0.04	0.35 ± 0.04	0.35 ± 0.05	0.33 ± 0.05 *†‡

*: *p* < 0.05 from SM BELT; †: *p* < 0.05 from SM BAR; and ‡: *p* < 0.05 from FW BAR.

**Table 5:** Agreement between SM BAR and FW BAR, SM BELT and FW BELT, SM BAR and SM BELT, and FW BAR and FW BELT. Typical error (TE), % Typical error (%TE), and Lin’s CCC at different loads.

	Velocity SM BAR and FW BAR			Velocity SM BELT and FW BELT			Velocity SM BAR and SM BELT			Velocity FW BAR and FW BELT		
Loads	TE	%TE	LIN’s CCC	TE	%TE	LIN’s CCC	TE	%TE	LIN’s CCC	TE	%TE	LIN’s CCC
30%	0.03	2.3%	0.95	0.04	3.0%	0.81	0.03	2.8%	0.83	0.013	1.0%	0.99
40%	0.02	2.0%	0.95	0.03	2.9%	0.82	0.03	2.8%	0.82	0.012	1.1%	0.99
50%	0.02	1.7%	0.96	0.03	2.7%	0.83	0.03	2.8%	0.81	0.011	1.1%	0.99
60%	0.01	1.6%	0.97	0.02	2.6%	0.85	0.02	2.8%	0.80	0.011	1.3%	0.98
70%	0.01	1.9%	0.95	0.02	2.8%	0.86	0.02	2.8%	0.86	0.011	1.5%	0.96
80%	0.02	3.0%	0.88	0.02	3.4%	0.85	0.02	2.8%	0.79	0.011	1.9%	0.93
90%	0.02	5.0%	0.71	0.02	4.8%	0.75	0.01	2.9%	0.81	0.012	2.5%	0.90
100%	0.03	8.7%	0.57	0.03	7.8%	0.61	0.01	3.1%	0.90	0.013	3.7%	0.89

**Table 6:** Pearson’s correlations between jump performance (SJ, CMJ, and DJ), load–velocity parameters, and strength measures (absolute and relative).

Variable	SJ	CMJ	DJ
V0 SM BAR	0.87 *	0.90 *	0.71 *
V0 SM BELT	0.74 *	0.77 *	0.67 *
V0 FW BAR	0.86 *	0.91 *	0.74 *
V0 FW BELT	0.89 *	0.93 *	0.80 *
L0 SM BAR	0.10	0.00	0.06
L0 SM BELT	0.05	−0.04	0.00
L0 FW BAR	0.36	0.24	0.39
L0 FW BELT	0.38	0.26	0.43
Maximum Strength SM	0.28	0.19	0.23
Maximum Strength FW	0.53	0.46	0.53
Relative Strength SM	0.47	0.34	0.43
Relative Strength FW	0.70 *	0.60	0.71 *

* and bold: *p* < 0.05.

**Table 7:** Pearson’s correlations between jump performance (SJ, CMJ, and DJ) and bar velocities across all loads in the SM BAR, SM BELT, FW BAR, and FW BELT conditions.

	Jump Performance vs. SM BAR Velocity			Jump Performance vs. SM BELT Velocity			Jump Performance vs. FW BAR Velocity			Jump Performance vs. FW BELT Velocity		
Loads	SJ	CMJ	DJ	SJ	CMJ	DJ	SJ	CMJ	DJ	SJ	CMJ	DJ
30%	0.90 *	0.91 *	0.75 *	0.78 *	0.79 *	0.76 *	0.88 *	0.92 *	0.75 *	0.91 *	0.93 *	0.81 *
40%	0.91 *	0.92 *	0.76 *	0.79 *	0.79 *	0.77 *	0.89 *	0.91 *	0.75 *	0.91 *	0.92 *	0.82 *
50%	0.91 *	0.92 *	0.78 *	0.79 *	0.79 *	0.79 *	0.90 *	0.90 *	0.75 *	0.91 *	0.91 *	0.82 *
60%	0.92 *	0.92 *	0.80 *	0.80 *	0.78 *	0.81 *	0.89 *	0.87 *	0.74 *	0.91 *	0.88 *	0.82 *
70%	0.92 *	0.90 *	0.82 *	0.78 *	0.76 *	0.82 *	0.85 *	0.80 *	0.69 *	0.87 *	0.82 *	0.79 *
80%	0.88 *	0.84 *	0.82 *	0.73 *	0.68 *	0.81 *	0.72 *	0.63 *	0.57	0.78 *	0.69 *	0.71 *
90%	0.71 *	0.65 *	0.72 *	0.54	0.46	0.68 *	0.45	0.31	0.32	0.55	0.42	0.51
100%	0.34	0.24	0.44	0.15	0.05	0.34	0.10	−0.06	0.03	0.21	0.05	0.21

* and bold: *p* < 0.05.

## Data Availability

The data are available upon request to the corresponding author.
